# Estrogen nuclear receptors affect cell migration by altering sublocalization of AQP2 in glioma cell lines

**DOI:** 10.1038/s41420-018-0113-y

**Published:** 2018-10-17

**Authors:** Shu Wan, Juanjuan Jiang, Chuanming Zheng, Ning Wang, Xia Zhai, Xiangwei Fei, Ruijin Wu, Xiuxiu Jiang

**Affiliations:** 1 Department of Neurosurgery, The First Affiliated Hospital, Zhejiang University School of Medicine, Hangzhou Shi, Zhejiang Province China; 2 Department of Otolaryngology, Hangzhou Children’s Hospital, Hangzhou City, Zhejiang Province China; 3 Key Laboratory of Head and Neck Cancer Translational Research, Department of Head and Neck Surgery of Zhejiang Cancer Hospital, Hangzhou City, Zhejiang Province China; 4 Department of Gynecology, Womens Hospital, Zhejiang University School of Medicine, Hangzhou City, Zhejiang Province China; 5 Cell-land Biology Technology Co., Ltd., Hangzhou City, Zhejiang Province China

**Keywords:** CNS cancer, Oncogenesis

## Abstract

Glioblastomas are capable of infiltrating into neighboring brain tissues. The prognosis of a male patient is worse than that of women. Here, we demonstrate the effects of estrogen on invasion of glioma cells via regulating estrogen nuclear receptors (ERα and ERβ) combined with aquaporin 2 (AQP2). In our study, we conclude that AQP2 was located mainly in the nuclei of the glioma cell lines and is capable of inhibiting cell invasion. According to the gene ontology analysis, out of 138 screened genes, three genes of ankyrin repeat and FYVE domain containing 1 (*ANKFY1*), lymphocyte transmembrane adaptor 1 (*LAX1*), and latent transforming growth factor beta-binding protein 1 (*LTBP1*) were found to be regulating the ERα and ERβ. The expression of ERα was found to be high, whereas the expression of both ERβ and AQP2 was low in glioma cells from patient tissues and glioblastoma cell lines. The expression levels of AQP2, ANKFY1, LAX1, and LTBP1 were upregulated by both ERα small interfering RNA (siRNA) and overexpression of ERβ. AQP2 inhibition of cell invasion was inversely influenced by LAX1siRNA. The luciferase report system indicated that AQP2 promoted the transcriptional activity of LAX1 and inhibited cell invasion. These data suggest that ERβ may function as AQP promoter in the nucleus to sustain cells' stability by promoting AQP production, while ERα acts as an antagonist of AQP2. The ratio between ERα and ERβ is likely to affect the distribution of AQP2 in the nucleus. Low level of ERβ reduces the inhibition of invasion of glioma cells influenced by high level of LAX1 expression, leading to an increase in the invasion ability of glioma cells.

## Introduction

Gliomas are the most common type of primary malignant brain tumors, having an overall poor prognosis. Evidence from a number of sources has suggested that female sex hormones play an important role in the development of gliomas in women^1^. Astrocytes express receptors for gonadal hormones and produce several neurosteroids, including estradiol (E2), progesterone, and testosterone^2^. Recent reports and clinical trials depicted the roles of estrogen receptor (ER) agonists in protecting the central nervous system from noxious consequences of neuroinflammation and neurodegeneration^3^. However, the role of estradiol in glioma migration and proliferation remains controversial. Malignant gliomas can infiltrate and spread widely into neighboring brain tissues. Sex steroids have been implicated in the development and progression of primary brain tumor^4^. For instance, androgen promotes glioma cell proliferation^5^, while progesterone promotes proliferation and invasion of glioma cells^6^. It is generally accepted that estrogen functions as a tumor promoter that induces cytoprotective mechanisms, which reduce apoptosis in medulloblastomas^7^. On the other hand, estrogens have a neuroprotective role in several neurological disorders, such as Parkinson’s disease, Alzheimer’s disease, and cerebrovascular accidents^8^. These neuroprotective effects include increased cell proliferation, cell migration, and inflammation. Cell migration is a highly orchestrated process. Estrogens promote neuronal differentiation, migration, and survival in the brain. Dysregulation of cell migration develops into tumor growth and metastatic processes. Due to intensive research findings regarding the role of E2 in promoting gliomas, the molecular mechanism of estrogen signaling cannot be ignored.

Aquaporins (AQPs) are small membrane channel proteins that control water and small solute transport through the phospholipid bilayers. AQPs are subdivided into water-selective channels and aquaglyceroporins^9^. In mammals, 13 isoforms (AQP0–AQP12) of the AQP protein family have been identified that have different properties of cellular localization and permeability for water. A growing body of evidence has suggested that AQPs could facilitate cell migration, invasion, and proliferation in tumor development with the aid of water transport^10,11^. Studies of tumor angiogenesis have revealed an unexpected function of AQPs in cell migration, invasion, and the spread of malignancy^12,13^. For instance, AQP1 increases cell migration and the metastatic potential of melanoma cells^14^. AQP4 is upregulated in astrocytomas and metastatic tumors, and the depletion of AQP4 reduces astrocyte cell migration^15,16^. In the reproductive system, AQP1, AQP5, and AQP8 have been shown to mediate E2-induced cell migration^17^. Our previous study also showed that AQP2 mediates E2-induced cell migration, invasion, and adhesion in endometrial cancer^18^. AQP2 was originally found in the collecting ducts of the kidneys, and it functions as a vasopressin-sensitive water channel in principal cells^19^. Our previous studies demonstrated that AQP2 was also expressed in the human endometrium and endometriosis. E2 might regulate the expression of AQP2 in endometrial cells^20,21^. However, the mechanism by which AQP2 mediates nervous system tumoral processes is still unclear. There is a lack of direct mechanistic evidence to explain AQP2 regulation of cell migration and invasion of glioma cells.

Therefore, it is indispensable to clarify the signaling responses triggered by E_2_ that are relevant to antiglioma effects. We aimed to investigate AQP2 involvement in estrogen-dependent glioma invasion to gain relevant mechanistic insights into this process. The experimental results from the present study provide several novel pieces of information regarding the mechanisms of AQP2 function and sublocalization in glioma by E2 and ERs. This work also provides us with the possibility for plausible antitumor therapy by targeting water channels in gliomas.

## Results

### Expression levels of AQP2 and ERs in human glioma cells

**Fig. 1:**
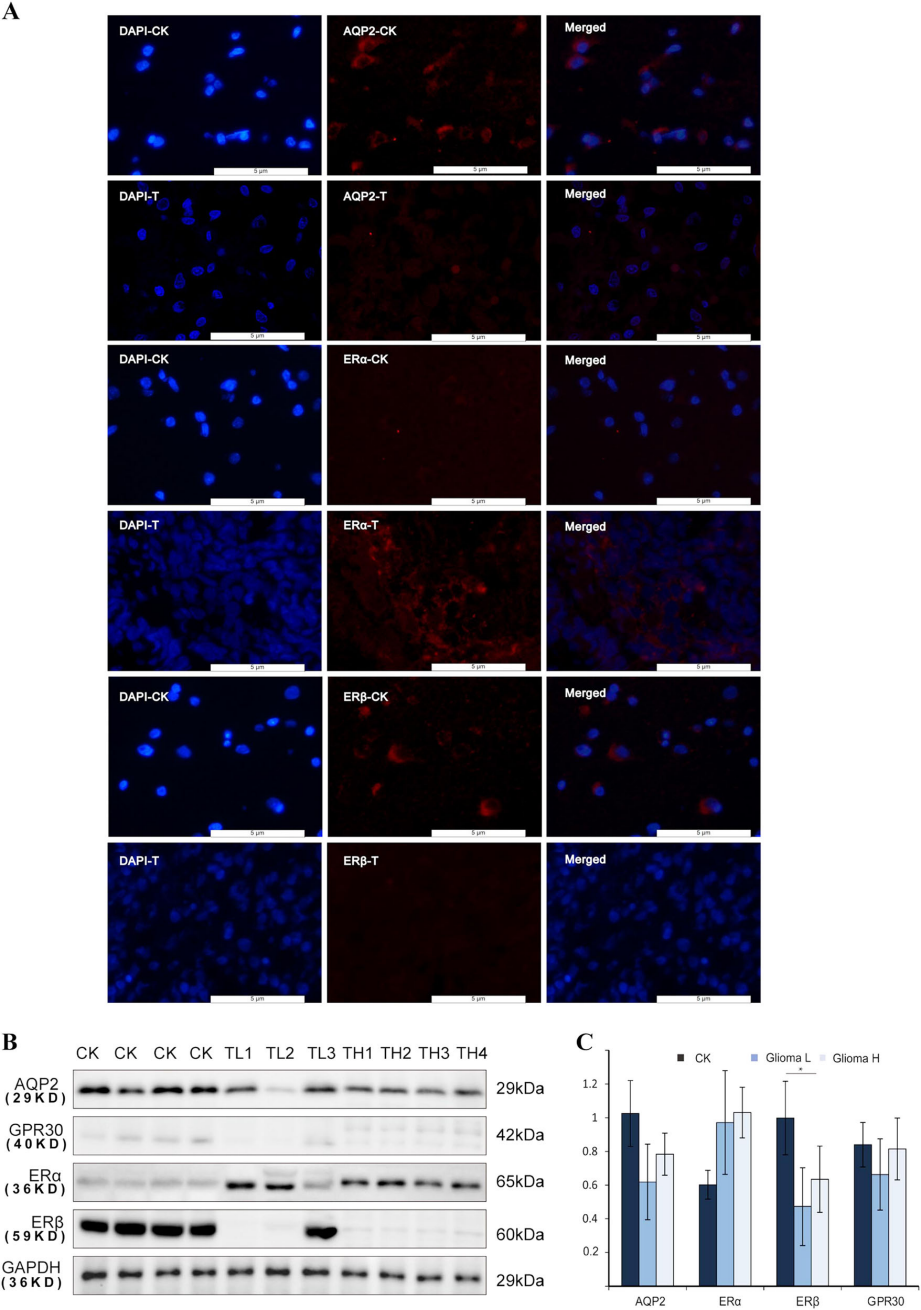
Expression of AQP2 and ERs in glioma and glial tissues from patients. Fluorescence staining analysis showed that AQP2, ERα, and ERβ were mainly located in the outer part of the nucleus (**a**). Target protein was stained in red. Cell nuclei were stained with DAPI. Western blot analysis demonstrated that the high expression levels of ERα, whereas low expression levels of ERβ and AQP2 were observed in glial cells compared to glioma cells (**b**, **c**). **P < 0.01 compared with the corresponding control. CK normal tissues, T tumor group, TL low-grade tumor, TH high-grade tumor

Based on the fluorescent staining (FS) results, we assume that AQP2 is located mainly in the outer part of the nuclei in glial and glioma cells in the tissues (Fig. 1a). Western blot analysis showed that expression levels of AQP2 were decreased in glioma cells compared to that in glial cells from the tissues (Fig. 1b). The expression levels of AQP2 in glioma cells from low-grade tumors (i.e., stages I and II) were lower in glioma cells than those in high-grade tumors (stages III and IV), but there was no statistical difference (Fig. 1b, c). It is generally accepted that ERα functions as a tumor promoter^22^, while ERβ functions as a tumor suppressor^23^. There was no report of the presence of GPR30 in the gliomas. In this study, we tested several ERs, including ERα, ERβ, and GPR30 in glioma and glial cells (Fig. 1b, c). ERα protein expression was higher in glioma cells than in glial cells. ERβ was mainly expressed in the astrocytes of low-grade gliomas and in normal astrocytes. ERβ levels were significantly decreased in glioma cells compared to those in glial cells. There was no significant difference in the expression of GPR30 between glioma and glial cells.

### E2 promotes cell migration and invasion via AQP2 in U87 cells

**Fig. 2:**
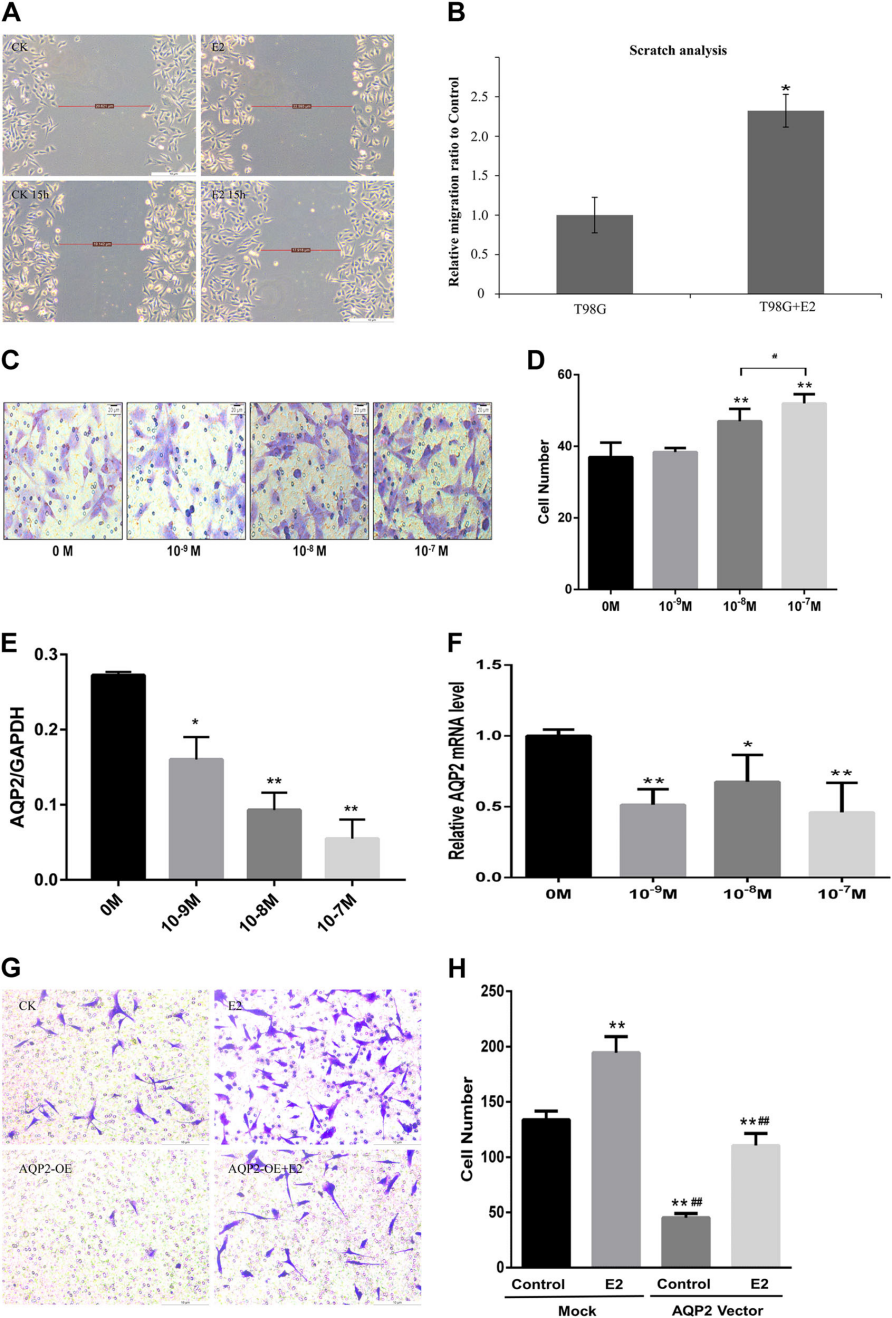
Effects of E2 on cell migration and invasion in T98G and U87 cell lines. Estrogen (E2) significantly increased the T98G cell migration (**a**, **b**) and U87 cell invasion capacity (**c**, **d**) in a concentration-dependent manner, as tested by wound scratch assay and transwell assay, respectively. Both T98G and U87 are glioblastoma cell lines. **e** RT-qPCR showed that E2 decreased the mRNA levels, and (**f**) western blotting showed decreased protein levels of AQP2 compared to the control. Overexpression of AQP2 decreased cell invasion, which was attenuated by E2 (**g**, **h**). Three independent experiments were repeated. **P < 0.01 vs. control; *^, #^*P* < 0.05 vs. control

To investigate the effects of E2 on cell migration and invasion, the T98G and U87 cell lines were treated with different concentrations (10^−9^, 10^−^^8^, or 10^−^^7^ M) of E2 dissolved by dimethyl sulfoxide for 24 h. Based on the results, it is proven that the relationship between E2 and both cell lines on invasion and migration is directly proportional, among which cell migration and cell invasion occur in an E2 concentration-dependent manner (Fig. 2a–d). To investigate the relationship between E2 and AQP2, notably, the protein (Fig. 2e) and mRNA levels of AQP2 were downregulated by E2 by 40–60% (Fig. 2f). Overexpression of AQP2 with the AQP2 vector (pSLLV-CMV-Zsgreen-puro) significantly decreased basal glioma cell invasion. Overexpression of AQP2 attenuated the stimulatory effect on cell invasion induced by E2 (10^−7^ M) (Fig. 2g, h). To investigate whether overexpression of AQP2 and AQP2 + E2 induced cell apoptosis, we conducted a cell proliferation experiment using CCK-8 solution and the result showed E2-promoted cell proliferation at a time-dependent but not concentration-dependent manner, while AQP2 had no effect on cell proliferation. These data meant that the decreased cell number in Fig. 2g was due to decreased cell migration number.

### E2 decreases intranuclear AQP2

**Fig. 3:**
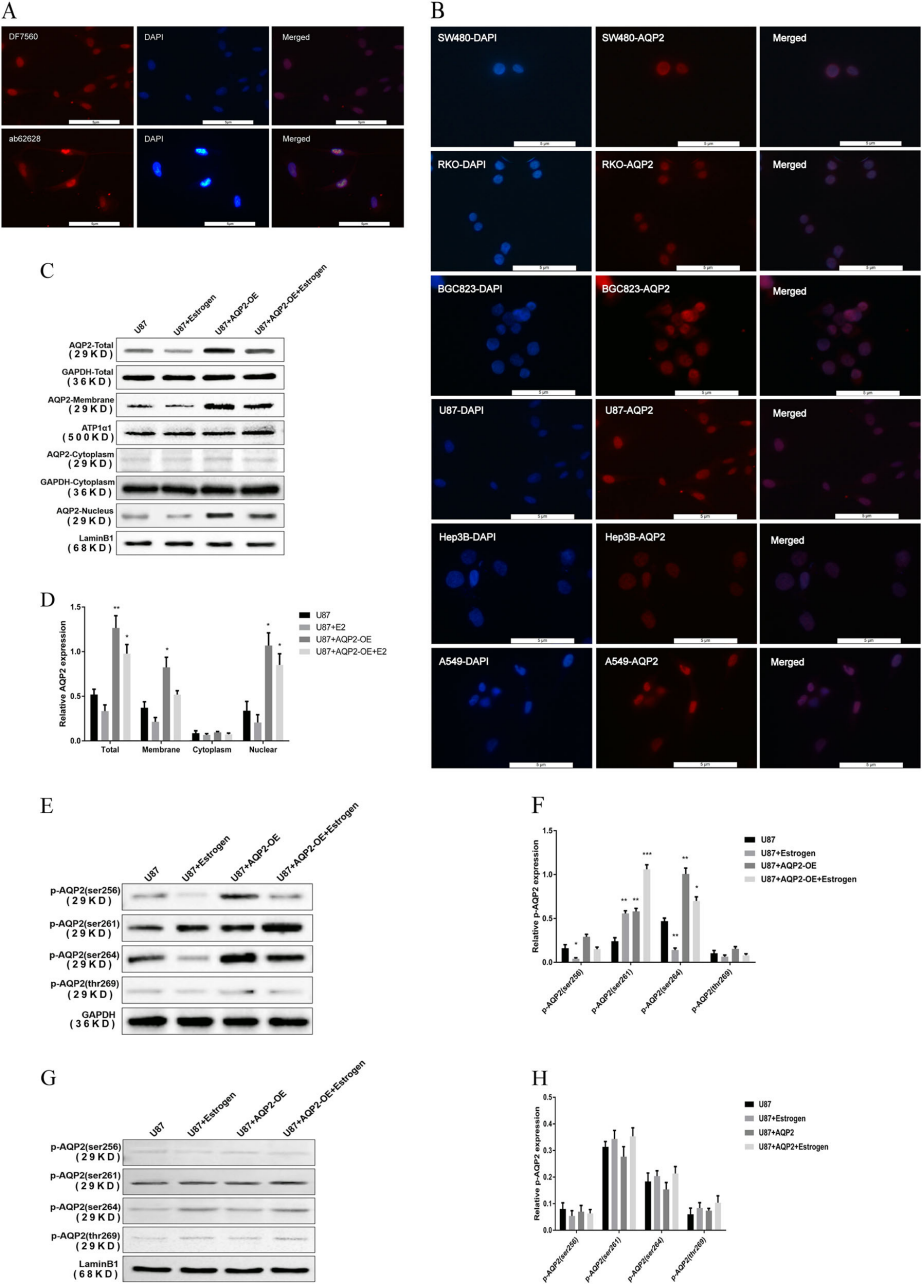
Effects of E2 on intranuclear AQP2 expression in tumor cell lines. **a** Experiments were repeated with two antibodies of AQP2 (DF7569 and Ab62628) in U87 cells. AQP2 was stained in red. Cell nuclei were stained with DAPI. **b** The phenomena of AQP2 sublocalization in the nuclei in cell lines were repeated and were confirmed in six other cell lines. **c**, **d** Western blot analysis of the cytoplasm and nucleus separately showed that E2 decreased total AQP2 expression in both the nucleus and cell membranes without change in the cytoplasm of U87 cells. **c**, **d** showed the results of RT-qPCR and western blotting, respectively. **e**, **f** E2 inhibited the total expression of p-AQP2 (ser256) and p-AQP2 (ser264) tested by western blotting and RT-qPCR, respectively. High expression level of both p-AQP2 (ser256) and p-AQP2 (ser264) observed in the AQP2 overexpression was attenuated by E2. The total expression level of p-AQP2 (ser261) was promoted by E2 and attenuated by overexpression of AQP2. ***P < 0.001 compared to control. **g**, **h** There was no obvious phosphorylation observed in the nucleus tested by western blotting and RT-qPCR, respectively. Three independent experiments were repeated. **P < 0.01 compared to control, *P < 0.05 vs. control. AQP2-membrane cell membrane, OE overexpression

To further investigate the sublocalization of AQP2 in glioblastoma cell lines, we performed FS in glioblastoma cell lines. The results demonstrated that AQP2 was located in the nucleus of the cell line; this finding was confirmed by two anti-AQP2 antibodies (Fig. 3a). This strange phenomenon was confirmed by six other malignant tumor cell lines (Fig. 3b). However, AQP2 was mainly expressed in the outer part of the nuclei in glial and glioma cells in the tissues (Fig. 1a). Based on GeneCards, AQP2 was supposed to locate mainly on the membrane. To further investigate the existence of a transmembrane mechanism of AQP2 in U87 cells, we performed a separation experiment of the cytoplasm and the nucleus. Under the treatment of 10^–7^ M E2 for 48 h, AQP2 expression was decreased both on the membrane and in the nucleus; however, there was no change observed in the cytoplasm (Fig. 3c, d). Thus, estrogen reduces the total amount of AQP2, rather than simply transferring AQP2 from the nucleus to the cell membrane. Since phosphorylated (p) AQP2 is the active form of AQP2^24^, does estrogen-induced cell migration be achieved by phosphorylation of AQP2 in the absence of a total AQP2? To study whether E2 decreases AQP2 accumulation on the membrane by inhibiting the phosphorylation of AQP2, the levels of p-AQP2 in the nucleus and in the whole cell were measured. By western blot analysis, p-AQP2 (ser256), p-AQP2 (ser261), p-AQP2 (ser264), and p-AQP2 (thr269) were detected. E2 inhibited phosphorylation of total AQP2. Increased total amount of p-AQP2 (ser261), but decreased p-AQP2 (ser256) and p-AQP2 (ser264), was observed with E2, which were attenuated by overexpression of AQP2 (Fig. 3e, f). There were no obvious changes in p-AQP2 (ser264) and p-AQP2 (thr269) content both in total and in the nuclei (Fig. 3e–h). These data indicated that p-AQP2 (ser256) and p-AQP2 (ser264) of total AQP2 were consistent with the AQP2 quantity changes on the membrane. These findings indicated that E2 reduces p-AQP2 (ser256) or p-AQP2 (ser264) content on the membrane, which may inhibit the accumulation of AQP2 on the membrane and promote cell migration.

### E2 influences intranuclear AQP2 function by regulating AQP2 downstream genes

**Fig. 4:**
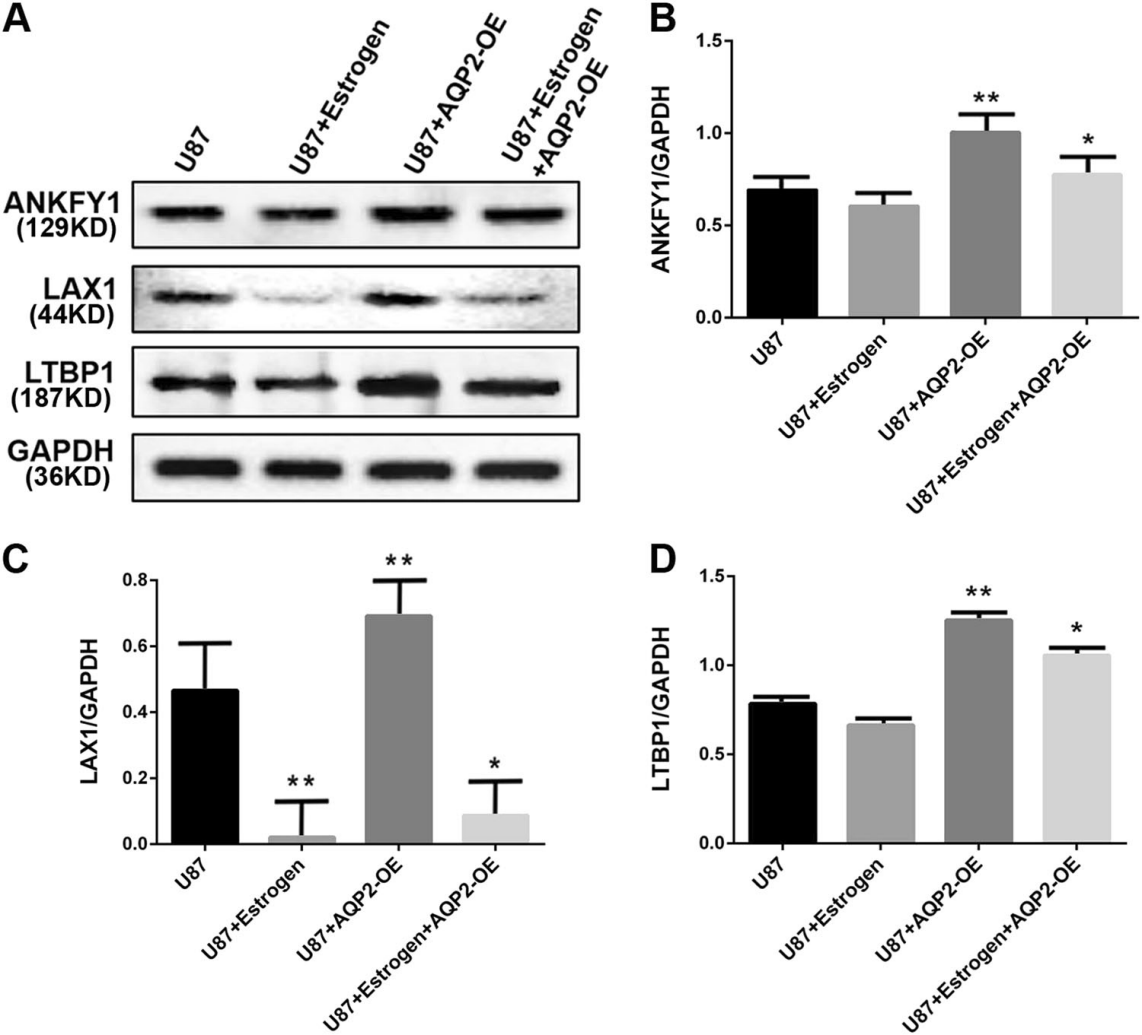
E2 influences AQP2 in the U87 cell nucleus regulated by *ANKFY1*, *LAX1*, and *LTBP1* genes. The expression of *ANKFY1*, *LAX1*, and *LTBP1* in U87 cell lines was analyzed by western blot (**a**) and RT-qPCR (**b**–**d**). E2 decreased the expressions of *ANKFY1*, *LTBP1*, and *LAX1* genes in nucleus of U87 cell line. Overexpression of AQP2 promoted the expressions of *ANKFY1*, *LAX1*, and *LTBP1* genes, whereas it was attenuated by E2. Three independent experiments were repeated. **P < 0.01 vs. control, *P < 0.05 vs. control. NC normal cells, AQP2NCsiRNA overexpression of AQP2

Since the role of estrogen in AQP2 in the nucleus is not achieved by phosphorylation, is there a downstream gene for AQP2 in the nucleus that affects cell migration and is regulated by estrogen? To investigate AQP2-bound downstream genes in the nucleus, we performed chromatin immunoprecipitation sequencing (ChIP-seq) with anti-AQP2-flag, collected DNA, and performed sequencing. Gene Ontology analysis showed enrichment for biological processes, cellular components, and molecular functions. One hundred and thirty-eight genes were recruited. Three genes related to tumor metastasis, namely, ankyrin repeat and FYVE domain containing 1 (*ANKFY1*), lymphocyte transmembrane adaptor 1 (*LAX1*), and latent transforming growth factor beta-binding protein 1 (*LTBP1*) (provided by RefSeq, Apr 2012), were selected for follow-up studies to test the effects of AQP2 on these genes. Overexpression of AQP2 significantly upregulated the mRNA and protein levels of *LAX1*, while E2 inhibited the regulatory effects of overexpression of AQP2 on *LAX1* (Fig. 4a–d). *LAX1* was chosen for further study.

### ERs bound to AQP2 function as *ANKFY1*, *LAX1*, and *LTBP1* gene promoters

**Fig. 5:**
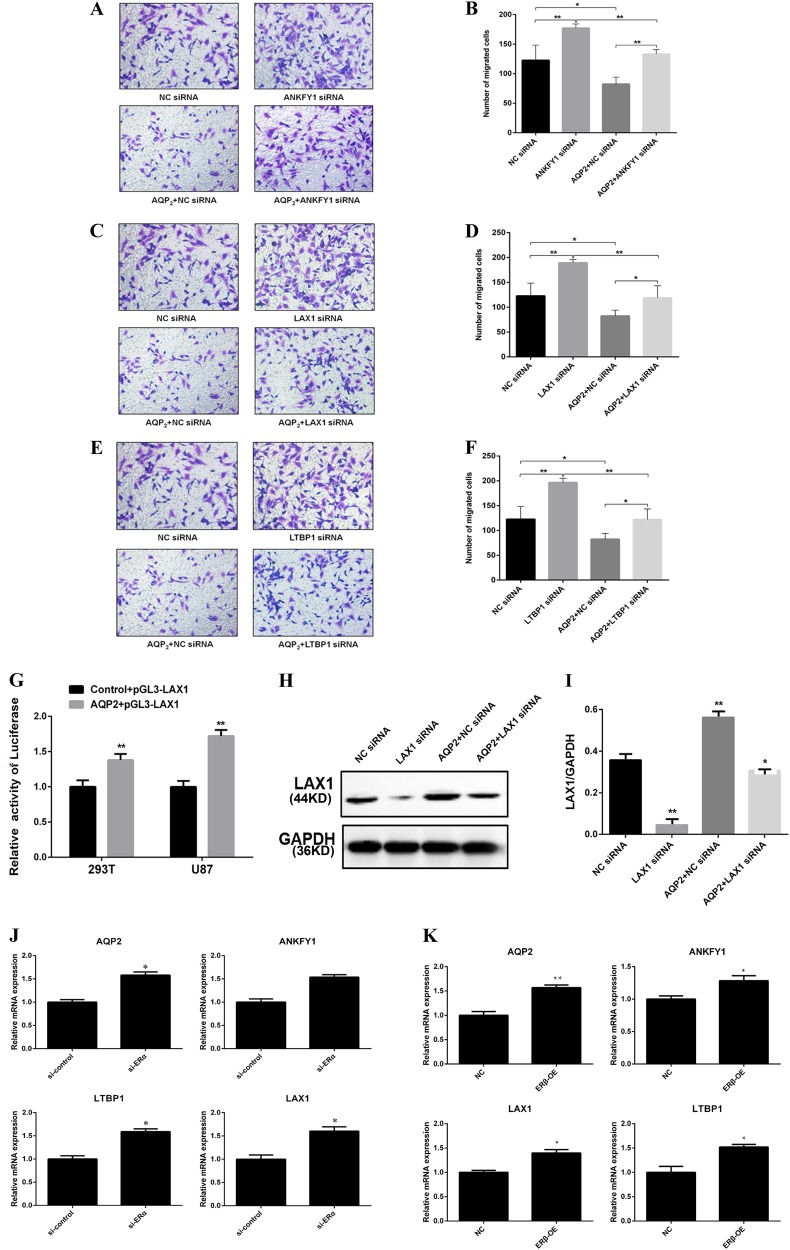
The pathway of E2 influences the localization of AQP2 in the U87 cell nucleus. Invasion of U87 cell was influenced by siRNA in relation to *ANKFY1*, *LAX1*, and *LETP1* genes analyzed using the transwell assay (**a**–**f**). Overexpression of AQP2 decreased the cell invasion, while it was attenuated by siRNA in relation to *ANKFY1*, *LAX1*, and *LETP1* genes. **g** showed that AQP2 + pGL3-LAX1 was loaded using HEK 293T vectors and transfected successfully to the U87 cell line. Luciferase reporter assays were performed. **h**, **i** Western blot and RT-qPCR showed *LAX1* gene expression in the nucleus. AQP2 promoted LAX1 expression, which was attenuated by LAX1siRNA. **j** showed that siRNA ERα increased ANKFY1, LAX1, LETP1, and AQP2 mRNA levels and was further corroborated by the overexpression of ERβ condition analyzed by RT-qPCR (**k**). The results are expressed as the means ± SEM of three independent experiments. *P < 0.05, **P < 0.01 vs. control. Si siRNA, AQP2NCsiRNA overexpression of AQP2

To determine how E2 influences cell invasion by cooperating with intranuclear AQP2, the relationship between ERs, AQP2, and the downstream genes was investigated. U87 cells were transfected with the corresponding gene small interfering RNA (siRNA). The transwell assay results showed that, after treatment with ANKFY1siRNA, LAX1siRNA, and LTBP1siRNA, respectively, the cell invasion capacities were promoted compared to control lentivirus (Fig. 5a–f). The *LAX1* gene was selected as an example to investigate LAX1 expression via regulation of AQP2 at the transcriptional level. After transfection with AQP2 + pGL3-LAX1 successfully (Fig. 5g), our results showed that overexpression of AQP2 increased LAX1 expression, while LAX1siRNA decreased AQP2 effects on LAX1 expression (Fig. 5h). AQ2 vector decreased cell invasion, while it was reversed by LAX1siRNA (Fig. 5c). Overexpression of ERβ upregulated the mRNA levels of ANKFY, LAX, LTBP, and AQP2, while ERαsiRNA increased the mRNA levels of ANKFY, LAX, LTBP, and AQP2 compared to those of the control groups (Fig. 5j, k). These data indicated that ERα and ERβ play an inverse influence on AQP2.

## Discussion

In this regard, the present study provided the following novel findings: (1) AQP2 expression was decreased in glioma cells in tissues; (2) AQP2 was located in all parts of glioma cells or glial cells in the tissues and was located mainly in the nuclei in cell lines; (3) E2 decreased AQP2 expression in cell lines, particularly in the nucleus; (4) E2 promoted cell invasion by reducing AQP2 distribution in the nucleus; and (5) the plausible molecular mechanisms by which E2 regulates AQP2 sublocalization and promotes cell invasion occurrence at the nuclear and membrane levels. E2 inhibited the phosphorylation of AQP2 of the membrane, which may reduce AQP2 accumulation on the membrane. At the nuclear level, AQP2-bound ERα/ERβ functioned as the promoters of *ANKFY1*, *LAX1*, and *LTBP1* genes.

The role of estrogen in glioma development remains controversial. Estrogens can exert their effects through intracellular or membrane-associated ERs, such as the intracellular receptors ERα/ERβ and GPRs. In this study, ERα protein expression levels were higher in glioma cells than in glial cells, while ERβ levels were significantly decreased in high-grade glioma compared with normal glial cells. This result was consistent with other reports that suggested that high expression of ERβ was an independent, favorable prognostic factor, but ERα was a poor prognostic factor in the multivariate analysis^25,26^. In this study, there was no significant difference in GPR30 expression between glioma cells and glial cells in the tissues. In addition to neurons and astrocytes, other cells, such as microglia and macrophage-like members of the intrinsic brain immune system, also express nuclear and nonnuclear ERs^27^. Experimental studies have shown that ERβ inhibits the proliferation of gliomas and induces cell death^28^. ERβ-selective agonists were found to inhibit the proliferation of glioma cell lines in vitro^29^. Thus, we inferred that the receptor quantity or ratio in astrocytic cells may influence E2 function and the prognosis of gliomas.

The underlying mechanisms of the regulation of AQP transcription via estrogen are complex. AQP2 forms a water-specific channel that provides the plasma membranes of renal collecting ducts with a high water permeability, thereby permitting water to move into the cells in the direction of an osmotic gradient. There have been no reports regarding AQP2 expression in gliomas. An important paralog of this gene is AQP5. It is known that phosphorylation of AQP5 results in internalization of the protein from the plasma membrane^30^. AQP5 showed dramatic adaptation to a changed environment and translocates into the nucleus by in vitro culture^31^. This is the precedent of the discovery of AQP2 with differential sublocalization in gliomas, with or without pretreatment with E2. Overexpression of AQP2 in the nuclei of U87 cells reduced cell invasion, suggesting the involvement of regulatory migration genes in this process. Upon binding of estrogen to an ER, the ligand receptor complex dimerizes and migrates into the nucleus, where the dimer binds to hormone response elements (HREs) in the promotor region of estrogen-responsive genes. Activation of the HRE leads to the induction or repression of gene transcription. Our ChIP sequence and luciferase reporter system indicated that AQP2-bound ERα/ERβ functioned as a promoter of *ANKFY1*, *LAX1*, and LTBP1 genes in the nucleus. In our previous study, we proved that there were EREs in the AQP2 promoter^18^. This finding supported that AQP2 might bind to ERα/ERβ and function as a promoter of *ANKFY1*, *LAX1*, and *LTBP1* genes in the nucleus. Furthermore, AQP2 promoted the transcription and expression of *ANKFY1*, *LAX1*, and *LTBP1* genes.

Phosphorylated AQP2 on the membrane provides a necessary and sufficient condition for the role of AQP2, while phosphorylation is not required for its function in the nucleus. According to the STRING database and group-based prediction system, AQP2 binds to protein kinase, cAMP-dependent, catalytic beta (PRKACB), directly, which was triggered by receptor binding to G-protein-coupled receptors. Protein kinase A activation regulates intracellular transport mechanisms and ion flux^32^. Moreover, GPR30 is one of the receptors of estrogen. Our findings showed that GPR30 was highly expressed on the membranes of glioma cells. E2 may bind to GPR30 and trigger PRKACB. This may inhibit phosphorylation of AQP2 also. In the present study, after E2 treatment, the changing trends in the total protein of p-AQP2 (ser256) and p-AQP2 (ser264) were consistent with the changing trend of AQP2 content on the cell membrane. As described in previous reports, there are reports of p-AQP2 (ser256), p-AQP2 (ser264), and AQP2 accumulation on the cell membrane^33^. Therefore, it is likely that E2 might reduce the accumulation of AQP2 on the membrane by lowering the content of p-AQP2. Thus, we concluded that E2 may reduce AQP2 accumulation on the membrane by inhibiting phosphorylation of AQP2 and reducing the quantity of AQP2 in the nucleus.

In short, our findings successfully proved that AQP2 regulation of glioma cell invasion was induced by E2. A novel transmembrane phenomenon in glioma cells was discovered. We proposed that E2 may reduce AQP2 accumulation on the membrane by inhibiting phosphorylation of AQP2 on the membrane and reducing the quantity and sublocalization of AQP2 in the nucleus.

## Materials and methods

### Subjects and sample collection

Ethical approval for this project was granted by the Ethics Committee of the First Hospital affiliated to the School of Medicine, Zhejiang University (No. 2018-500). Written informed consent was obtained from each subject before tissue collection. The glial samples were collected from patients (*n* = 4, two women and two men, median age = 47 years; range, 40–55 years) who attended to the Department of Neurosurgery of the First Hospital Affiliated of School of Medicine, Zhejiang University for brain injuries and were obtained by biopsy for diagnostic purposes. The glioma samples were collected from patients (*n* = 4, two women and two men; median age, 50 years; range, 44–67 years) who underwent surgical treatment for glioma (International Federation of Gynecology and Obstetrics (FIGO)) stage III and IV patients (*n* = 3, two women and one man; median age, 46 years; range, 43–48 years) and who underwent surgical treatment for glioma (FIGO) stage I and II. None of these patients received preoperative radiation or chemotherapy. Diagnosis of glioma was pathologically confirmed. No patients had received hormone therapy in the prior 3 months.

### Cell culture condition

The U87 cell line, derived from male glioblastomas, was obtained from the American Type Culture Collection (Manassas, VA, USA, ATCC HTB-14, USA). The T98G cell line, derived from male glioblastoma multiforme, was obtained from China Shanghai Zeye Biological Technology Co., Ltd. The p3B2.1-7 cell line, derived from human liver cancers; the RKO cell line, derived from human colonic cancer; the A549 cell line, derived from human non-small-cell lung cancer; the SW480, derived from human colonic cancer; and the BGC823, derived from human gastric cancer were all obtained from the Chinese Academy of Sciences, Shanghai Life Science, Cell Resource Center. The cell lines were cultured as previously described^34^.

### RNA extraction and qRT-PCR

Total RNA was extracted and purified using the Universal RNA Extraction Kit (MiniBEST, TaKaRa, Japan) according to the manufacturer’s instructions. Real-time PCR amplifications were conducted in triplicate. The primer sequences used for quantitative real-time PCR (qRT-PCR) are listed in Table 1.

**Table 1:** Sequences of the primers used for real-time PCR

Gene	Forward (5′–3′)	Reverse (5′–3′)
*AQP2*	CGTTTGGCTTGGGTATTGGC	CGGATGTCTGCTGGCGTGA
*AQP4*	GAGTGACAGACCCACAGCAAGG	AAAGCAAAGGGAGATGAGAACC
*AQP5*	GGCTGCCATCCTTTACTTCTACCT	GCTCCTCCCAGTCCTCGTCA
*AQP8*	CGTTCTCCATCGGCTTTGC	AGGCGGGTCTTCCCATCTC
*ANKFY1*	TAGCACCATTTGAAATCAGTGTT	TGGTGTCGTGGAGTCG
*LAX1*	CGCAAGGATGACACGCAAATTC	TGGTGTCGTGGAGTCG
*LTBP1*	GTCCTGGTGGAATGGGTTATAC	GTTGAGTGTTCTTTGGCTTGAC
*LARP1*	GTTTCCTACCCATCACCCTTATT	CACCACCTTGCTGTCCTTTA
*RFC3*	GGCTCATAGACTTGCAGAGAAG	CCCAATCTGTCTCAGGGATTTC
*siRNA NC*	UUCUCCGAACGUGUCACGUTT	ACGUGACACGUUCGGAGAATT
*ANKFY1 siRNA1*	GCUGCAGUGCAAACAACUATT	UAGUUGUUUGCACUGCAGCTT
*ANKFY1 siRNA2*	GGACUUCAUUUGAUGAGAATT	UUCUCAUCAAAUGAAGUCCTT
*ANKFY1 siRNA3*	GCCCAUGUCAACCACAGAATT	UUCUGUGGUUGACAUGGGCTT
*LAX1 siRNA1*	GGAAUUGGAAUAAACGGAATT	UUCCGUUUAUUCCAAUUCCTT
*LAX1 siRNA2*	GCAUACAGCCCACAUCCAUTT	AUGGAUGUGGGCUGUAUGCTT
*LAX1 siRNA3*	GCUGAGACUCUAGCUUCUATT	UAGAAGCUAGAGUCUCAGCTT
*LTBP1 siRNA1*	GCCAUCUUCCAUGUAUGAATT	UUCAUACAUGGAAGAUGGCTT
*LTBP1 siRNA2*	GCCUAAACUUUAUCAGCAUTT	AUGCUGAUAAAGUUUAGGCTT
*LTBP1 siRNA3*	GCCAAUCCCAAGUCUCGUATT	UACGAGACUUGGGAUUGGCTT
*ESR1shRNA*	GAGGGAGAAUGUUGAAACATT	UGUUUCAACAUUCUCCCUCTT
*ESR1shRNA*	GGUUCCGCAUGAUGAAUCUTT	AGAUUCAUCAUGCGGAACCTT
*ESR1shRNA*	GGCUAGAGAUCCUGAUGAUTT	AUCAUCAGGAUCUCUAGCCTT
*GAPDH*	ACTCCCATTCTTCCACCTTTG	CCCTGTTGCTGTAGCCATATT

### Western blot analysis

For western blotting, cells were lysed in RIPA buffer (Beijing Solarbio Science and Technology Co., Ltd., Beijing, China).

### Transwell migration assay

Cell culture inserts were seeded with 5 × 10^5^ of the cell lines. The U87 and T98G cells were treated with different concentrations (10^−9^, 10^−^^8^, or 10^−^^7^ M) of E2 (Sigma-Aldrich, E8875). Cells on the lower aspect of the mounted membranes were viewed and photographed under a microscope every 24 h. For quantification, the number of stained cells in five selected areas (top, middle, bottom, left, and right) was manually counted.

### Wound scratch assay

Different groups of cells were grown in serum-free culture to confluence in a six-well plate. After 12 h, the cells were wounded, and the healing capability was calculated by evaluating the amount of cell coverage to the scratch zone at 0 and 24 h.

### Fluorescent staining

Cells were blocked with Dako Protein Block (Dako, Mississauga, ON, Canada) as previously described^18^. The AQP2 antibody (1:100 diluted in Dako Protein Block, DF7560, Affinity Co., Cincinnati, OH, USA), the corresponding secondary donkey anti-rabbit IgG H&L (1:100 diluted in Dako Protein Block, Alexa Fluor 647), another AQP2 antibody (1:500 diluted in Dako Protein Block, Ab62628, Abcam Co., USA), the corresponding secondary goat anti-rabbit IgG H&L (1:10,000 diluted in Dako Protein Block, BL003A, Biosharp), anti-ERα (1:100 diluted in Dako Protein Block, Proteintech, 21244.1.AP), rabbit anti-ERβ mouse (1:100 diluted in Dako Protein Block, Santa Cruz Sc-53494), and horseradish peroxidase-conjugated secondary antibodies (MultiSciences GAR007, GAM007) were used. Finally, the cells were counterstained with the chromosomal dye 4′,6-diamidino-2-phenylindole (DAPI) (Beyotime Biotechnology, C1006).

### Separation of the nucleus and cytoplasm

According to the manufacturer’s instructions (Membrane Nuclear and Cytoplasmic Protein Extraction Kit, Sangon Biotech, China; C510002), protein from the nucleus, cytoplasm, and membrane was obtained.

### RNA interference experiments

ANKFY1, LAX1, LTBP1, and ER-α (ESR1) siRNA duplexes were chemically synthesized by Ambion (Carlsbad, CA, USA). Silencer FAM-labeled negative siRNA was used as a control oligonucleotide. The primer sequences used for the qRT-PCR are listed in Table 1.

### Lentiviral vector construction and transfection

Lentivirus vectors expressing flag-tagged AQP2 and ERβ (HG11302-ACG) were generated by inserting the corresponding complementary DNA into the multicloning site of the lentivirus backbone plasmid of pSLLV-CMV-Zsgreen-puro and Pcmv3-C-GFPSpark, respectively. The constructs were co-transfected with packaging vectors into 293T cells for packaging, which was followed by purification (Cell-Land Biological Technology, Hangzhou, China).

### Bioinformatics analysis and ChIP

The primer sequences used to amplify the precipitated DNA are listed in Table 1. Target gene sequences were constructed from the NCBI databases (http://www.ncbi.nlm.gov/gene). The pCDH-CMV-MCS-EF1-copGFP-AQP2 vector with the AQP2-flag fusion protein was constructed, and the AQP2 lentivirus packaging was successful and highly infected. ChIP-seq was performed as previously described^35^.

### Plasmid construction and luciferase reporter assay

To predict AQP2 binding to the DNA of the LAX1 promoter at conserved sites, we constructed a luciferase reporter plasmid with the LAX1 promoter ligated to the luciferase expression vector pGL3. DNA fragments of human LAX1 promoters were amplified from NCBI after being compared with the original LAX1 sequence, and the vector was constructed successfully. The luciferase values were normalized to the 293T + pGL3-Basic or U87 + pGL3-Basic vector.

### Statistics

Multiple comparisons were analyzed by one-way or two-way analysis of variance, followed by multiple comparison tests using the SPSS 19.0 software (SPSS, Chicago, IL, USA). Statistical significance was defined as *P* < 0.05.

## Electronic supplementary material

Supplementary data
