# Supplementary figures and images for: Estrogen nuclear receptors affect cell migration by altering sublocalization of AQP2 in glioma cell lines
Source: Cell Death Discov. 2018 Oct 17;4:49. doi: 10.1038/s41420-018-0113-y (PMC6192986; doi:10.1038/s41420-018-0113-y)

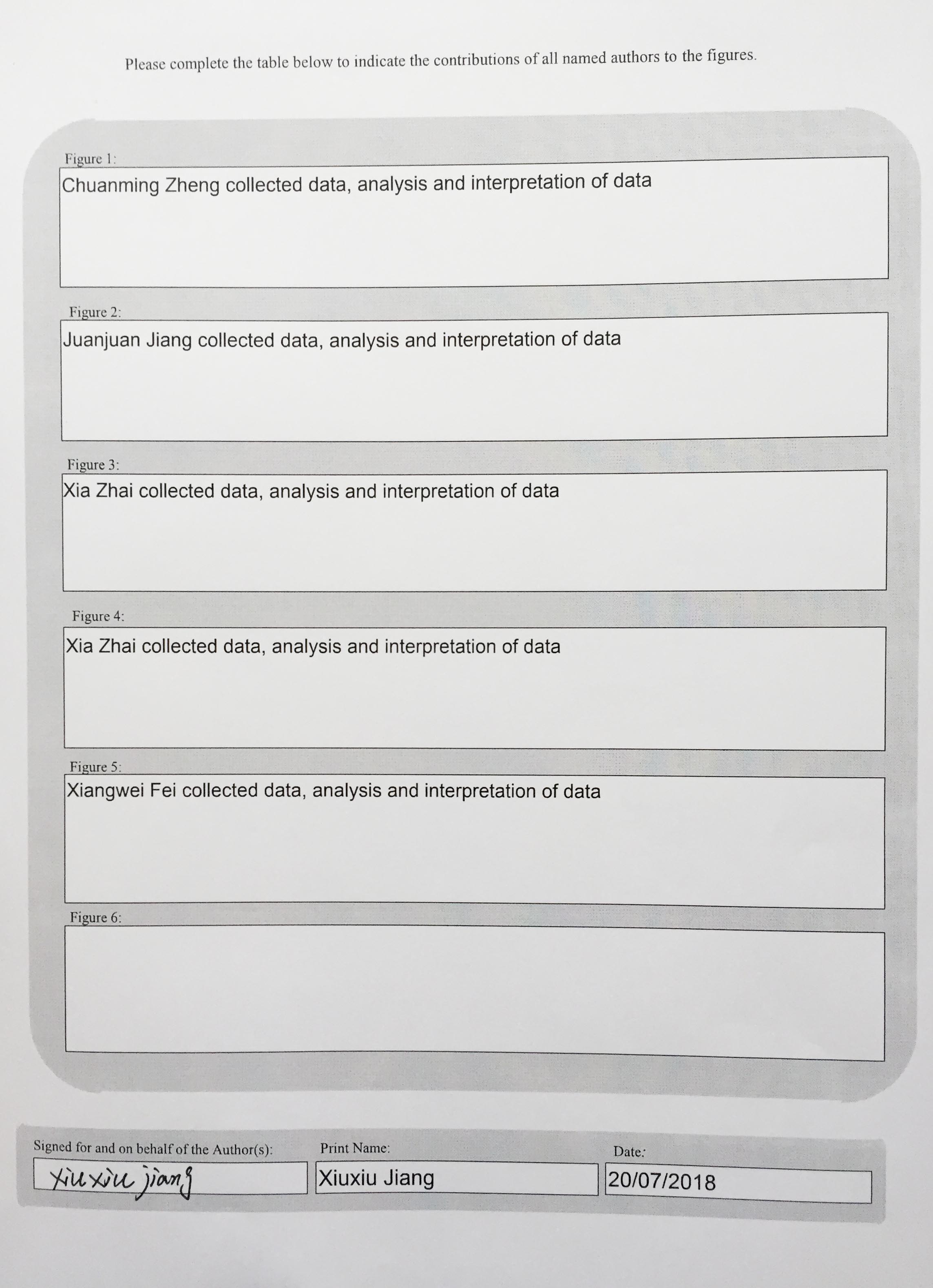

Supplement: Supplementary file 1 — Supplementary data [file 41420_2018_113_MOESM1_ESM.jpg]
